# Time-Dependent and Organ-Specific Changes in Mitochondrial Function, Mitochondrial DNA Integrity, Oxidative Stress and Mononuclear Cell Infiltration in a Mouse Model of Burn Injury

**DOI:** 10.1371/journal.pone.0143730

**Published:** 2015-12-02

**Authors:** Bartosz Szczesny, Attila Brunyánszki, Akbar Ahmad, Gabor Oláh, Craig Porter, Tracy Toliver-Kinsky, Labros Sidossis, David N. Herndon, Csaba Szabo

**Affiliations:** 1 Department of Anesthesiology, The University of Texas Medical Branch, Galveston, TX, United States of America; 2 Shriners Hospitals for Children, Galveston, TX, United States of America; 3 Department of Surgery, The University of Texas Medical Branch, Galveston, TX, United States of America; 4 Department of Biochemistry and Molecular Biology, The University of Texas Medical Branch, Galveston, TX, United States of America; Faculty of Medicine & Health Sciences, UNITED ARAB EMIRATES

## Abstract

Severe thermal injury induces a pathophysiological response that affects most of the organs within the body; liver, heart, lung, skeletal muscle among others, with inflammation and hyper-metabolism as a hallmark of the post-burn damage. Oxidative stress has been implicated as a key component in development of inflammatory and metabolic responses induced by burn. The goal of the current study was to evaluate several critical mitochondrial functions in a mouse model of severe burn injury. Mitochondrial bioenergetics, measured by Extracellular Flux Analyzer, showed a time dependent, post-burn decrease in basal respiration and ATP-turnover but enhanced maximal respiratory capacity in mitochondria isolated from the liver and lung of animals subjected to burn injury. Moreover, we detected a tissue-specific degree of DNA damage, particularly of the mitochondrial DNA, with the most profound effect detected in lungs and hearts of mice subjected to burn injury. Increased mitochondrial biogenesis in lung tissue in response to burn injury was also observed. Burn injury also induced time dependent increases in oxidative stress (measured by amount of malondialdehyde) and neutrophil infiltration (measured by myeloperoxidase activity), particularly in lung and heart. Tissue mononuclear cell infiltration was also confirmed by immunohistochemistry. The amount of poly(ADP-ribose) polymers decreased in the liver, but increased in the heart in later time points after burn. All of these biochemical changes were also associated with histological alterations in all three organs studied. Finally, we detected a significant increase in mitochondrial DNA fragments circulating in the blood immediately post-burn. There was no evidence of systemic bacteremia, or the presence of bacterial DNA fragments at any time after burn injury. The majority of the measured parameters demonstrated a sustained elevation even at 20–40 days post injury suggesting a long-lasting effect of thermal injury on organ function. The current data show that there are marked time-dependent and tissue-specific alterations in mitochondrial function induced by thermal injury, and suggest that mitochondria-specific damage is one of the earliest responses to burn injury. Mitochondria may be potential therapeutic targets in the future experimental therapy of burns.

## Introduction

Severe burn injury results in an inflammatory and hypermetabolic stress leading to profound alterations in energy expenditures. Even though the burn insult, itself, is a localized event, the response of the body is systemic; it triggers, among others, hepatic dysfunction, cardiac dysfunction, skeletal muscle dysfunction, pulmonary alterations and disturbances in metabolism and growth and development; many of these alterations extend well beyond the time of the healing of the burn wound (months to years) [1–4].

A key event in the pathogenesis of thermal injury is the excess generation of reactive oxygen/nitrogen species (ROS/RNS) produced by a variety of cellular sources including the mitochondria [5–7]. One of the downstream effects of ROS/RNS-mediated damage is the induction of DNA damage, including various base lesions and single-strand breaks (SSBs), in both nuclear and the mitochondrial DNA. Mitochondrial DNA is more susceptible to ROS-induced damage than its nuclear counterpart, yet mitochondrial DNA damage, mitochondrial dysfunction and its consequences have, so far, received little attention in connection with the pathophysiological effects of burn injury. Also, although mitochondrial dysfunction has been linked with the hypermetabolic stress response to burn injury [8–10], the role of mitochondrial DNA damage in response to thermal trauma has not yet been thoroughly investigated.

Taking into account that mitochondria generate 90% of cellular ATP, it is important to carefully define the role of the mitochondria in the pathogenesis of burn trauma. In the present study we investigated the changes in several mitochondrial functions in response to scald burn injury in mice. We observed a differential degree of remote tissue damage, as measured by the presence of lipid peroxidation products (malondialdehyde, MDA), as well as marked changes in mitochondrial bioenergetics and DNA damage to the mitochondrial and nuclear DNA, with the highest levels of damage observed in the lungs and the heart. The observed damage persisted for approximately 40 days post-injury. We also detected a marked increase of circulating mitochondrial DNA fragments early after injury (with a peak at 3 hours) suggesting that mitochondria may be particularly sensitive to thermal injury, which is consistent with the observed remodeling of mitochondrial bioenergetics.

## Materials and Methods

### Mouse Model of Burn Injury

All animal procedures were performed according to the National Institutes of Health guidelines for experimental animal use. Permission to perform this study was obtained from the Institutional Animal Care and Use Committee at the University of Texas Medical Branch in Galveston. Male BALB/c mice (10–12 weeks old) were housed at 24–26°C on a 12:12 light:dark cycle. Sham and burn treated mice underwent identical experimental procedures, with the exception of injury. Following an intraperitoneal (i.p.) injection of 0.1 mg/kg buprenorphine, mice were anesthetized by inhalation of 3–5% isoflurane. Next, ~40% of the dorsum was shaved with electrical clippers and ~ 1cc of lactated ringers (LR) solution was injected under the skin along the spinal column. The dorsa of burn treated animals were then exposed to ~95°C water for 10 sec to produce a full thickness scald wound covering ~30% of the total body surface area (TBSA). Mice were then resuscitated with 2 cc of LR. Burn and sham treated mice were individually housed throughout the experimental period. To minimize animal suffering, pain or distress, animals were scored twice daily throughout the post burn period using an IACUC-approved Rodent Intervention Score Sheet to assess their well-being and clinical status by certified veterinarian. To minimize suffering of the animals the analgesic buprenorphine was administered when indicated to reduce pain and distress. Cohorts of mice were sacrificed at 3 hours, 1, 4, 10, 20, and 40 days post-injury. Heart, liver and lung tissues were harvested for further analysis.

### Mitochondrial Isolation for Bioenergetics Studies

Mitochondria from freshly excised livers and lungs of sham and burn animals were immediately isolated by differential centrifugation [9,10]. The mitochondrial isolation buffer (MSHE + BSA) was composed of 210 mM mannitol, 70 mM sucrose, 5 mM HEPES, 1 mM EGTA and 0.5% (w/v) fatty acid-free BSA, pH 7.2. The mitochondrial assay solution (MAS-1) was composed of 220 mM mannitol, 70 mM sucrose, 10 mM KH_2_PO_4_, 5 mM MgCl_2_, 2 mM HEPES, 1 mM EGTA and 0.2% (w/v) fatty acid-free BSA, pH 7.2. All steps of mitochondria isolation were performed at 4°C. Briefly, tissues were extracted and minced in 10 volumes of MSHE + BSA. The tissue was homogenized with a drill-driven Teflon glass homogenizer with 8–10 strokes. Homogenate was centrifuged at 600 × g for 10 min, the supernatant was decanted to a separate tube and centrifugation was repeated 2 more times. Next, the supernatant was transferred to a new tube and centrifuged at 10,000 x g for 10 min, the supernatant fraction was eliminated and the fat/lipid layer, which covered the crude mitochondria pellet was carefully aspirated. The pellet was resuspended in 2 ml of MSHE + BSA, and the centrifugation step was repeated 2 more times at 10,000 × g for 10 min. Finally, the pellet was resuspended in 100 μl of MSHE + BSA. Protein concentration (mg/ml) was determined using DC Protein Assay from BioRad. Mitochondrial preparations were used within 1–4 h for functional assays.

### Measurement of Mitochondrial Bioenergetics

The XF24 Extracellular Flux Analyzer (Seahorse Biosciences, North Billerica, MA) was used to measure mitochondrial bioenergetic function, as described earlier [9,10]. Briefly, respiration by the mitochondria (5 μg/well) was sequentially measured in a coupled state with succinate (5.5 mM) as a substrate (basal respiration, State 2), followed by State 3 (phosphorylating respiration, in the presence of ADP (4 mM), State 4 (non-phosphorylating or resting respiration) following conversion of ADP to ATP and State 4o, induced with the addition of oligomycin (2 μM). Next, maximal uncoupler-stimulated respiration (State 3u) was detected by the administration of the uncoupling agent FCCP (4 μM). At the end of the experiment the Complex III inhibitor antimycin A (4 μM) was applied to completely inhibit mitochondrial respiration. Inclusion of rotenone (2.2 μM) next to succinate in the initial condition (in state 2) triggers the respiration to be driven only by Complex II–IV. This ‘coupling assay’ examines the degree of coupling between the electron transport chain (ETC), and the oxidative phosphorylation (OXPHOS), and can distinguish between ETC and OXPHOS with respect to mitochondrial function/dysfunction. Since we measured mitochondrial bioenergetics of isolated mitochondrial from various time points after injury (ranging from 3 hours to 40 days), we analyzed at least 2 shams and 6 burn injured animals per each time point; mitochondria from sham and burn animals was always included on the same Seahorse plate, in order to minimize inter-assay variability. Due to lack of statistical significant differences between the mitochondrial function of the various sham animals, we present the sham samples studied at various time points after burn together as a single sham group.

### Quantification of DNA Damage

Integrity of the nuclear and the mitochondrial DNA, measured by the relative amount of DNA damage, was analyzed by semi-quantitative, long-amplicon PCR assays (LA-PCR) using LongAmp Taq DNA Polymerase (New England BioLabs, Ipswich, MA) [11]. Total DNA was isolated using DNase Blood and Tissue Kit (QIAGEN, Hilden, Germany). Briefly, damage to nuclear DNA was estimated by quantification of the PCR amplification of the 10kb nuclear-specific DNA fragment using PicoGreen fluorescent dye to detect amplified double-stranded DNA (Quant-iT^™^ PicoGreen; Life Technologies, Carlsbad, CA). Damage to the mitochondrial DNA was estimated by quantification of the PCR amplification of the 8.9kb mitochondrial-specific DNA fragment using PicoGreen staining. Obtained data was normalized by the secondary PCR amplification of 221bp mitochondrial genome-specific fragment for correction of the multiple copies of the mitochondrial DNA. LA-PCR assay is based on premises that DNA damage inhibits progression of DNA polymerase during PCR reaction and thus amplification of appropriate DNA fragment is negatively correlated with the level of the DNA damage.

### Measurement of Malondialdehyde

Concentration of the most abundant lipid peroxidation product, malondialdehyde (MDA), was measured with ALdetect (MDA-Specific) Lipid Peroxidation Assay Kit (Enzo Life Science, Farmingdale, NY) according to the manufacturer’s recommendation. The final MDA concentration is expressed in μM.

### Mitochondrial Volume (Citrate Synthase Activity)

Citrate synthase, the initial enzyme of TCA cycle and marker of the mitochondrial matrix is frequently used as a normalization factor for various mitochondrial-specific activities, and was determined using a Citrate Synthase Assay Kit according to the manufacturer’s recommendations (Sigma).

### Myeloperoxidase Activity

Tissue infiltration by polymorphonuclear leukocytes (neutrophils and monocytes) as an indication of inflammation was detected by measurement of myeloperoxidase (MPO) activity using Myeloperoxidase Fluorometric Detection Kit according to the manufacturer’s recommendation. The final MPO activity is express as U/g of wet tissue.

### Quantification of Tissue Poly of ADP-Ribose (PAR) Groups

To determine the activity of PARP after burn injury, we measured the amount of PARylation in liver, lungs and hearts in homogenates obtained 3 hours, 24 hours, 4 days and 10 days post burn, together with their sham controls. PAR groups were detected using PAR-specific antibody (BD Bioscience) and ECL Western Blotting substrates (Pierce). Densitometry analysis of signal was performed using a Gel Imaging system (G:Box, Syngen).

### Quantification of Circulating Mitochondrial DNA in Plasma

Total DNA content of mice plasma was determined by spectrophotometry and diluted to 100 ng/μl. For the determination of plasma mtDNA content, Real-time qPCR based method was performed using CFX96 Touch^™^ Real-Time PCR Detection System (Bio-Rad) and Maxima^™^ SYBR green qPCR master mix (ThermoScientific) with the following primer set: 5’-CCG AGC TAC TAC CAT CAT TCA AGT-3’; 5’-GAT GGT TTG GGA GAT TGG TTG ATG T-3’, which give amplicon of 117bp. Next, mtDNA content was calculated in 100 ng of total plasma DNA by ddCT method. Calculated data were normalized to sham plasma mtDNA content in 100 ng of total plasma DNA and represented in %. In order to assess the specificity of the mtDNA primers and, in order to rule out bacterial infection induced by burn injury, we perform similar experiments using total DNA isolated from mouse liver and three most frequently occurring upon infection bacterial strains, namely, *Escherichia coli*, *Pseudomonas aeruginosa* and *Staphylococcus aureus*.

### Bacterial Cultures

To investigate infection induced by burn injury blood and burn wounds or non-injured skin from the same area of sham-injured mice were harvested at 1 and 4 days post-burn or sham injury. Wounds and skin were homogenized in sterile saline, and 0.1 ml blood and homogenates was plated on tryptic soy agar plates and incubated overnight at 37°C for determination of colony forming units (cfu) per gram of wound/skin or ml of blood.

### Histopathology and Immunohistochemistry

4 mice of each experimental group (sham and burn) were sacrificed at 24 hours and 4 days post burn/sham injury. Heart, lung and liver tissues were harvested for histological analysis using hematoxylin & eosin staining and for immunohistochemical localization of MPO (abcam#9535), CD68, a marker of marker for the various cells of the macrophage lineage (including monocytes, histiocytes, giant cells, Kupffer cells, and osteoclasts) (abcam#125212) and neutrophil elastase (abcam#21595).

### Statistical Analysis

Data are shown as means standard deviation (SD). Student’s t-test was used to detect differences between groups; *p<0.05 and **p<0.01 represent statistically significant differences.

## Results

### Burn Injury Induces Temporal Changes in Mitochondrial Bioenergetics

**Fig 1:**
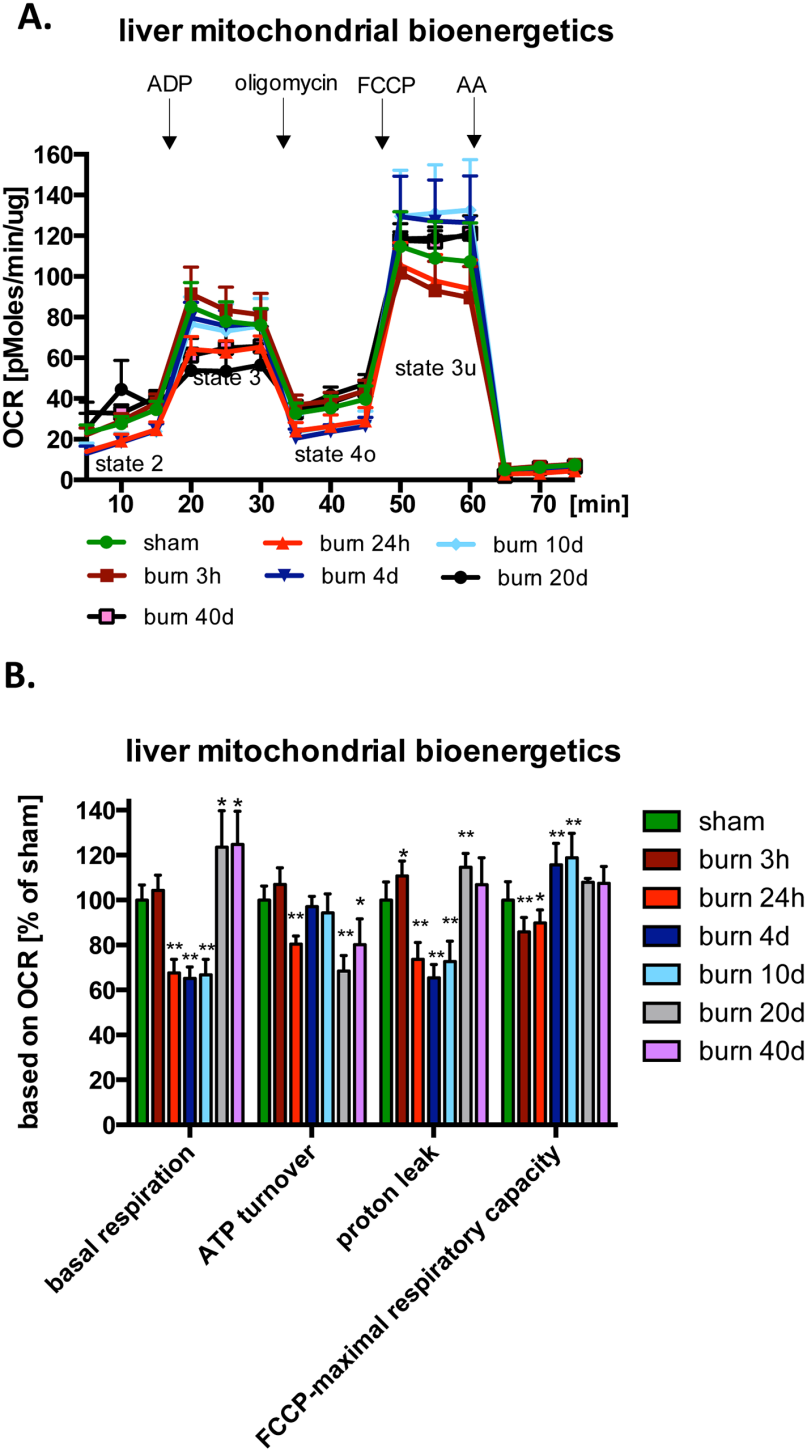
Burn injury induces time-dependent changes in the mitochondrial bioenergetics in the liver. Bioenergetic parameters of the isolated mitochondria from liver of mice subjected to burn injury were analyzed by Extracellular Flux Analysis. **(A)** Traces of oxygen consumption of mitochondria isolated from sham or burn injured animals at various time points post burn injury are shown. **(B)** Calculated bioenergetics parameters are shown. The results are based on n = 6 per group and represent mean±SD. *, ** p<0.05 and p<0.01 respectively, relative to sham animals.

**Fig 2:**
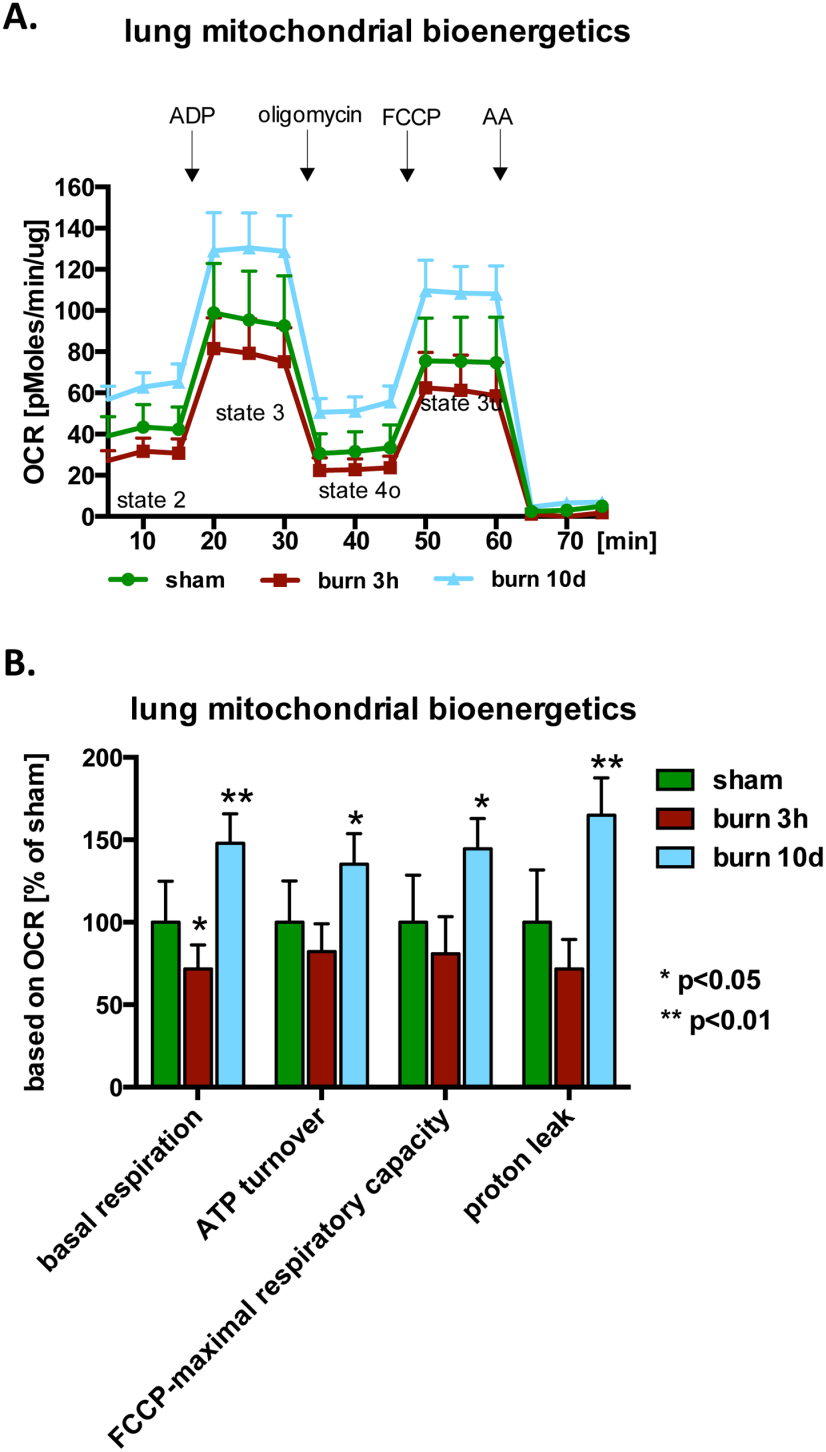
Burn injury induces time-dependent changes in the mitochondrial bioenergetics in the lung. Bioenergetic parameters of the isolated mitochondria from lungs of mice subjected to burn injury were analyzed by Extracellular Flux Analysis. **(A)** Traces of oxygen consumption of mitochondria isolated from sham or burn injured animals at various time points post burn injury are shown. **(B)** Calculated bioenergetics parameters are shown. The results are based on n = 6 per group and represent mean±SD. *, ** p<0.05 and p<0.01 respectively, relative to sham animals.

The effect of burn injury in changes of major bioenergetics parameters was investigated with the Extracellular Flux Analysis method in mitochondria isolated from the liver and the lungs at various time points post-burn. A significant decrease of major bioenergetic parameters; namely, basal respiration, ATP synthesis and maximal respiratory capacity, was detected at the early time points post injury in the liver mitochondria (Fig 1A and 1B). Although proton leak and maximal respiratory capacity returned to normal by the end of the study, some other mitochondrial functional parameters remained altered even at 40 days: basal respiration remained higher than control, while ATP turnover remained lower than control (Fig 1B). Significant changes in the mitochondrial bioenergetic parameters were also detected in mitochondria isolated from the lung tissue at 3 hours and 10 days post-burn (Fig 2A and 2B). We detected a reduction in basal respiration, ATP-synthesis and maximal respiratory capacity linked oxygen consumption as early as 3 hours post-burn (Fig 2B). However, at 10 days post-burn, all measured bioenergetic parameters were elevated, when compared to sham treated animals (Fig 2B). Taken together, our data indicate that similar changes occur in the mitochondrial bioenergetics of both tissues analyzed, with an initial decrease of major parameters in response to burn injury, followed by a (perhaps compensatory) increase in some of the bioenergetic parameters studied.

### Burn Induces Tissue-Specific, Persistent DNA Damage

**Fig 3:**
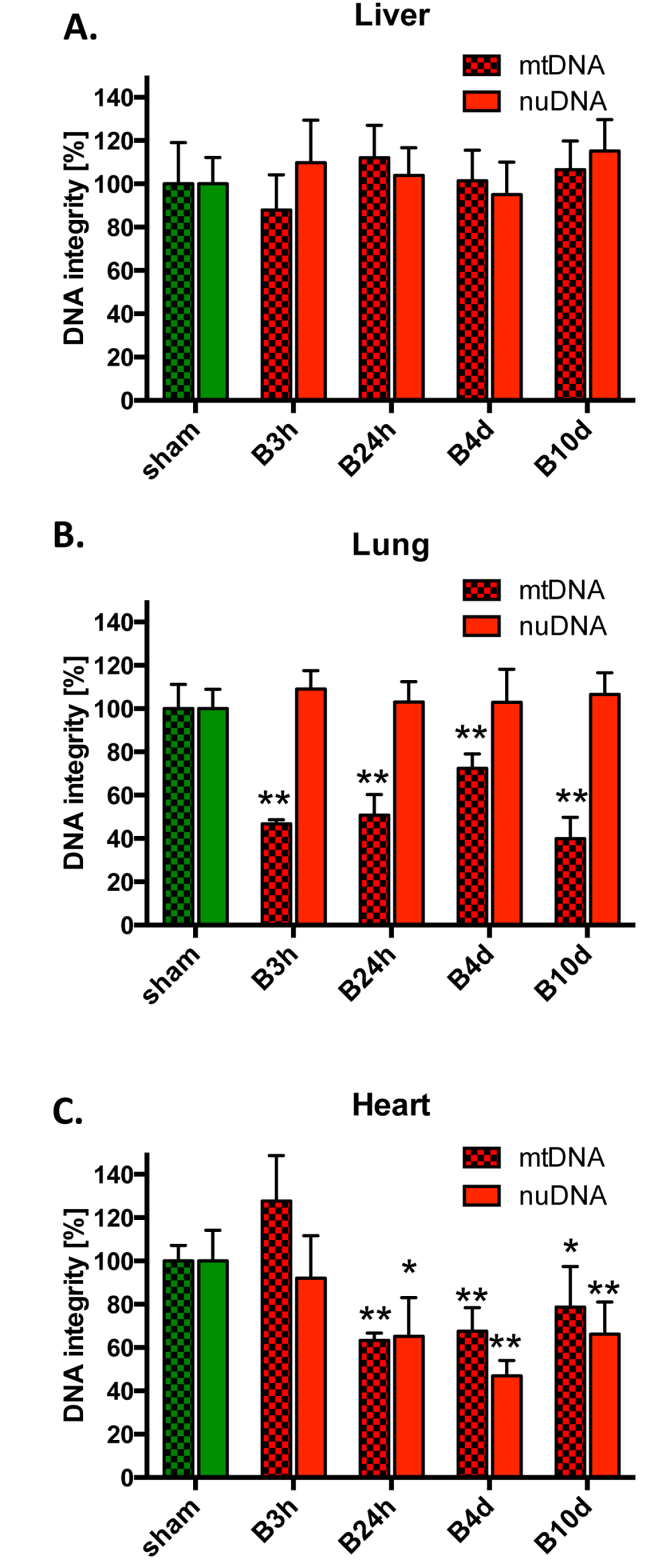
Changes in mitochondrial and nuclear DNA integrity in response to burn injury. Damage to nuclear and mitochondrial DNA was quantified using LA-PCR assay in **(A)** liver, **(B)** lungs and **(C)** heart of mice subjected to burn injury. The results are based on n = 6 per group and represent mean±SD. *, ** p<0.05 and p<0.01 respectively, relatively to sham animals.

**Fig 4:**
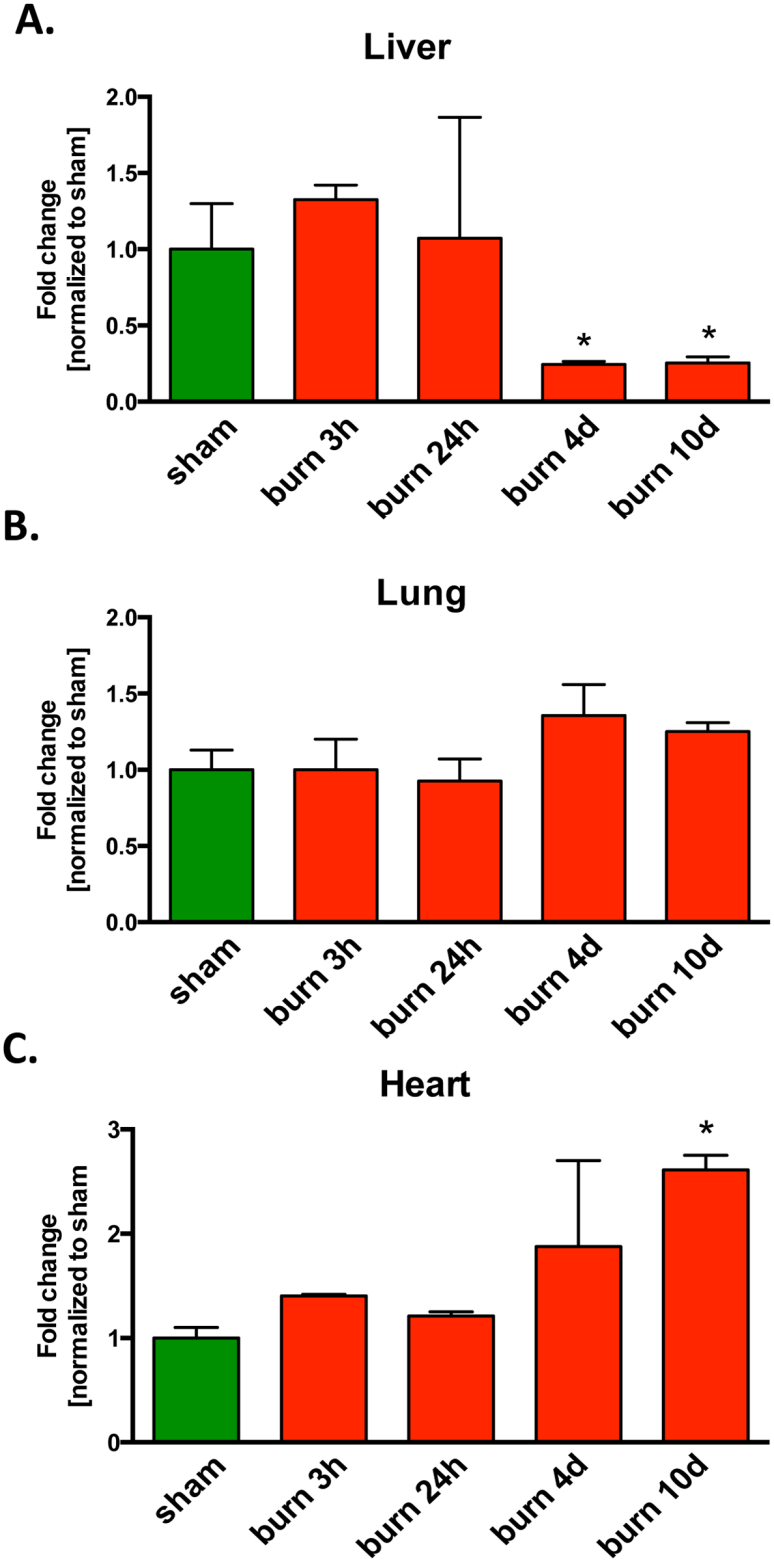
Changes in the amount of poly(ADP-ribose) (PAR) groups in response to burn injury. PARylation in total cell extracts was analyzed in **(A)** liver, **(B)** lungs and **(C)** heart of mice subjected to burn injury and compared to sham treated animals. The results are based on n = 4 per group and represent mean±SD. *p<0.05, relatively to sham treated animals.

We have also investigated the effect of burn injury on the DNA damage in three organs: the liver, the lungs and the heart. The integrity of mitochondrial and nuclear DNA, as reflected by the amount of DNA damage at various times post-burn injury was estimated using PCR of long DNA fragments (LA-PCR assay), the only currently available technique for concurrent measurement of DNA damage in both genomes [11]. This assay detects mostly DNA breaks, single- and double-strand DNA breaks, thus it underestimates the degree of DNA damage (since DNA polymerase by-passes most DNA lesions, e.g. those induced by oxidative stress, such as 8-oxoguanine) [12]. Although, we were not able to detect significant changes in the integrity of the mitochondrial and nuclear DNA in the liver after burn in comparison to sham treated animals (Fig 3A), the mitochondrial DNA integrity in the lungs was reduced by 50% as early as 3 hours post-burn (Fig 3B). Interestingly, mitochondrial DNA damage in the lung tissue persisted even at 10 days post-burn, while no detectable damage was observed to the nuclear DNA in the same organ (Fig 3B). Damage to both mitochondrial and nuclear DNA was observed in the heart at 24 h and the damage persisted until 10 days post-injury (Fig 3C). Since the accumulation of DNA breaks after oxidative/nitrative stress may either result in the activation of PARP enzymes and/or the degradation of PARP via caspases and other intracellular proteases, we followed our DNA damage analysis by measuring amount of poly-ADP-ribose groups in tissue extracts of burn injured animals at various time points (Fig 4). Burn injury resulted in a decrease of the PARylation in liver at 4 and 10 days post injury as compared to sham treated animals (Fig 4A) consistently with our previous observation about lack of burn induced DNA damage in liver tissue (Fig 3A). In, contrast, an increase in PARylation was detected in the heart at 4 and 10 days post burn (Fig 4C), which is in line with our observation demonstrating the accumulation of persistent DNA damage in heart tissue upon burn injury (Fig 3C). Taken together, these data indicate that there is a tissue-specific accumulation of DNA damage induced by burn, with the heart and the lungs being more affected than the liver, which results in the activation of PARP, and, consequently, increased accumulation of PAR polymers in the heart, but not in the liver.

### Mitochondrial Volume Decreases at the Early Time Points after Burn Injury

**Fig 5:**
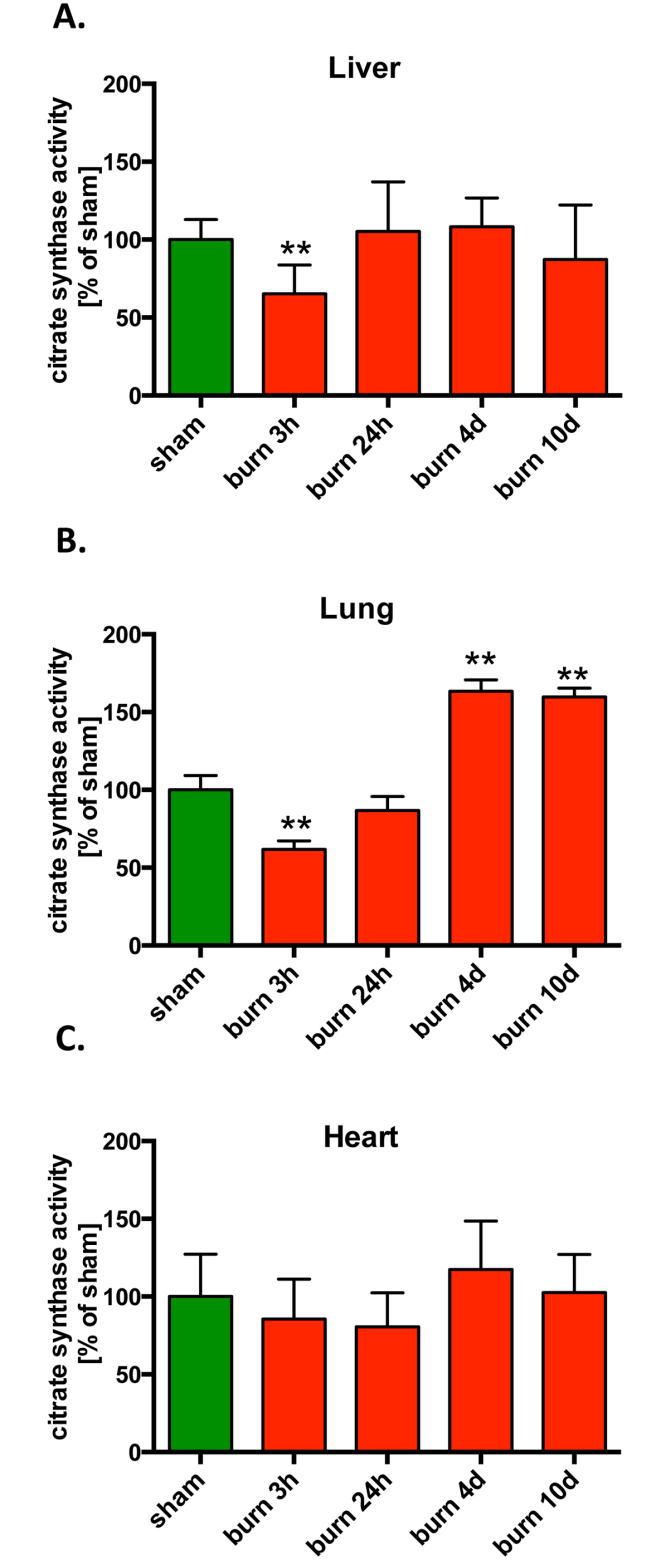
Changes in mitochondrial volume in response to burn injury. Mitochondrial biogenesis was assayed using specific activity of citrate synthase in extract isolated from **(A)** liver, **(B)** lungs and **(C)** heart of mice subjected to burn injury. The results are based on n = 6 per group and represent mean±SD. ** p<0.01, relatively to sham animals.

Since we observed significant changes in mitochondrial bioenergetics and mitochondrial DNA integrity after burn injury, next we evaluated whether burn also induces changes in the mitochondrial biogenesis. Citrate synthase, a commonly used quantitative marker for mitochondrial mass, was measured in liver, lung and heart after burn injury. We observed a significant decrease in citrate synthase activity at 3 hours post-burn in the liver and the lungs (Fig 5A and 5B), with no detectable changes in later time points in the liver. However, a 50% increase in mitochondrial volume was noted in the lungs at 10 days post-burn (Fig 5C), suggesting that increased mitochondrial biogenesis may be a potential mechanism utilized by the lungs to compensate for the negative effect of burn injury. In contrast to the changes noted in the lung, no significant changes in citrate synthase activity were noted in the heart tissue in response to burn injury.

### Burn Induces the Accumulation of Lipid Peroxidation Products, Particularly in the Heart

**Fig 6:**
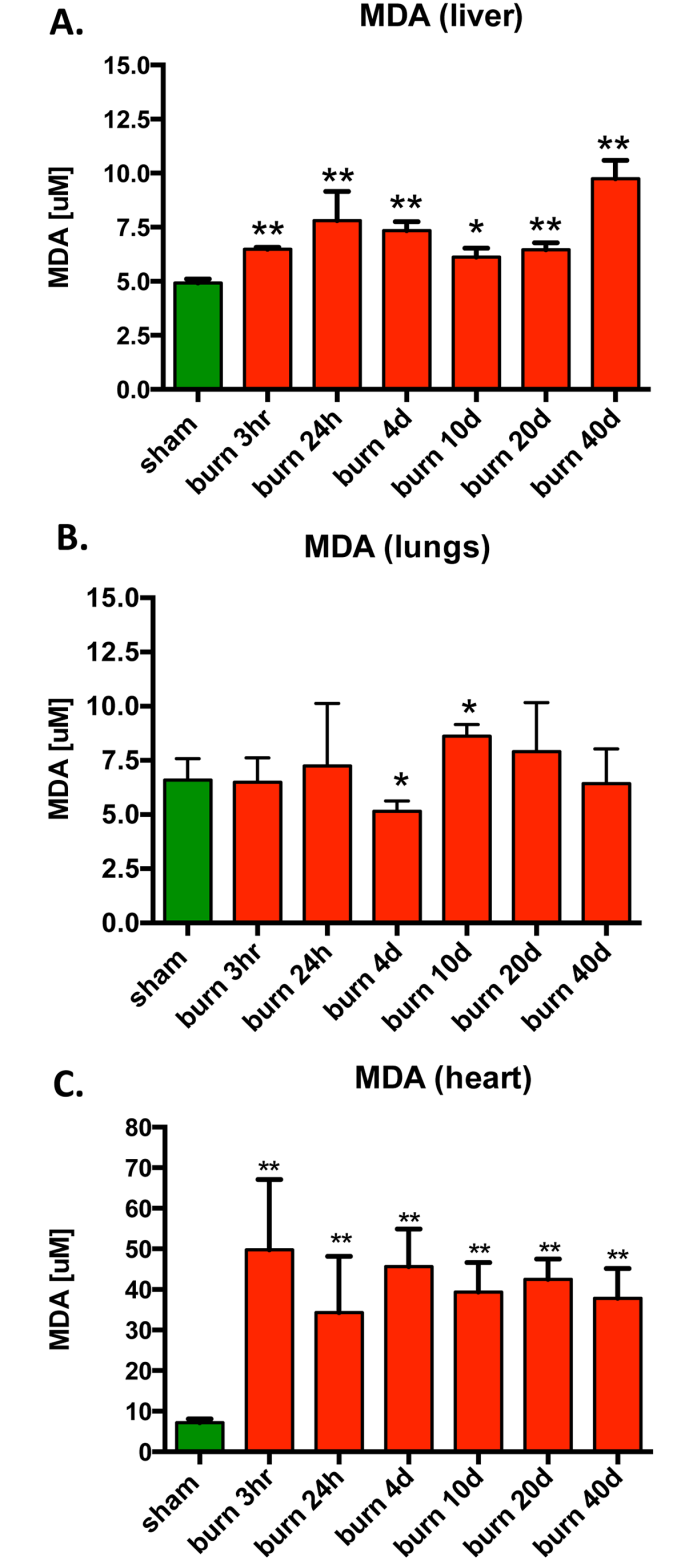
Changes in MDA, an index of lipid peroxidation, in response to burn injury. MDA levels were measured in **(A)** liver, **(B)** lungs and **(C)** heart of mice subjected to burn injury. The results are based on n = 6 per group and represent mean±SD. *, ** p<0.05 and p<0.01 respectively, relatively to sham animals.

Burn injury is known to be associated with increased oxidative stress. We have measured the levels of the most abundant lipid peroxidation product, malondialdehyde (MDA) in the lungs, liver and heart tissues of the animals subjected to burn injury. Although we detected a statistically significant increase of the MDA in the liver and lung at various post-burn time points as compared to sham-treated animals (Fig 6A and 6B), the changes were relatively modest. In contrast, a 10-fold increase in MDA levels was detected in the heart of animals subjected to burn injury; this increase manifested as early as 3 hours post-injury and persisted even at 40 days post-burn (Fig 6C), suggesting a long-lasting oxidative stress in this organ following burn injury.

### Burn Induces a Time-Dependent Accumulation of Myeloperoxidase in Lung and Heart

**Fig 7:**
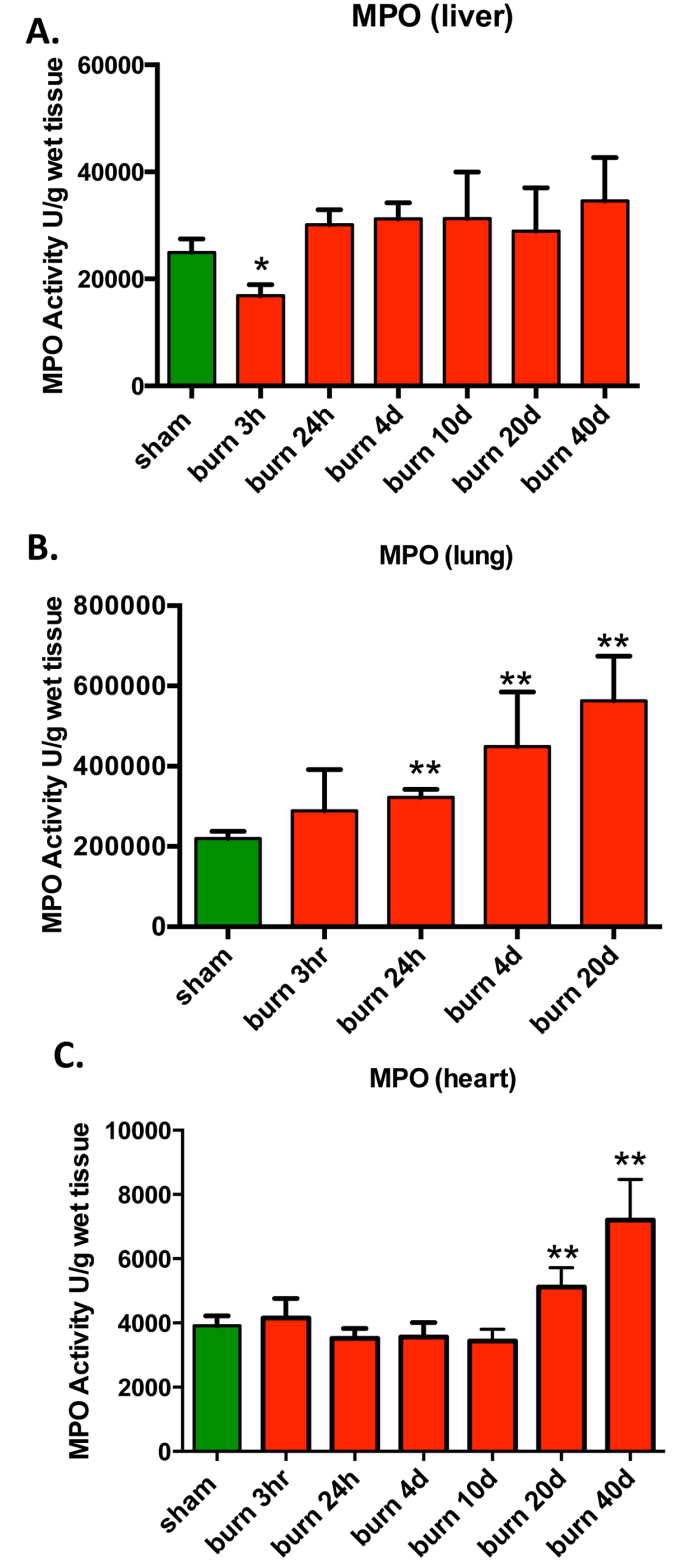
Changes in MPO, an index of mononuclear cell infiltration, in response to burn injury. MPO levels were measured in **(A)** liver, **(B)** lungs and **(C)** heart of mice subjected to burn injury. The results are based on n = 6 per group and represent mean±SD. *, ** p<0.05 and p<0.01 respectively, relatively to sham animals.

Oxidative stress and inflammation are intimately intertwined in many pathophysiological condition, because oxidants can upregulate pro-inflammatory signal transduction pathways, and the infiltration of activated mononuclear and polymorphonuclear cells into tissues can elevate oxidant production. Here we investigated the amount of myeloperoxidase (MPO), an enzyme stored in azurophilic granules of polymorphonuclear neutrophils and macrophages, in tissue homogenates as a marker of inflammatory cell infiltration into the parenchymal organs after burn injury. The heart and the lung, but not the liver showed a time-dependent increase in MPO activity post-burn (Fig 7). Elevated MPO activity persisted even at 40 days post-burn. In the lung and heart we observed a 2-3-fold increase of MPO at 20 and 40 days post burn (Fig 7B and 7C).

### Burn injury Is Associated with the Release of DNA into the Circulation

**Fig 8:**
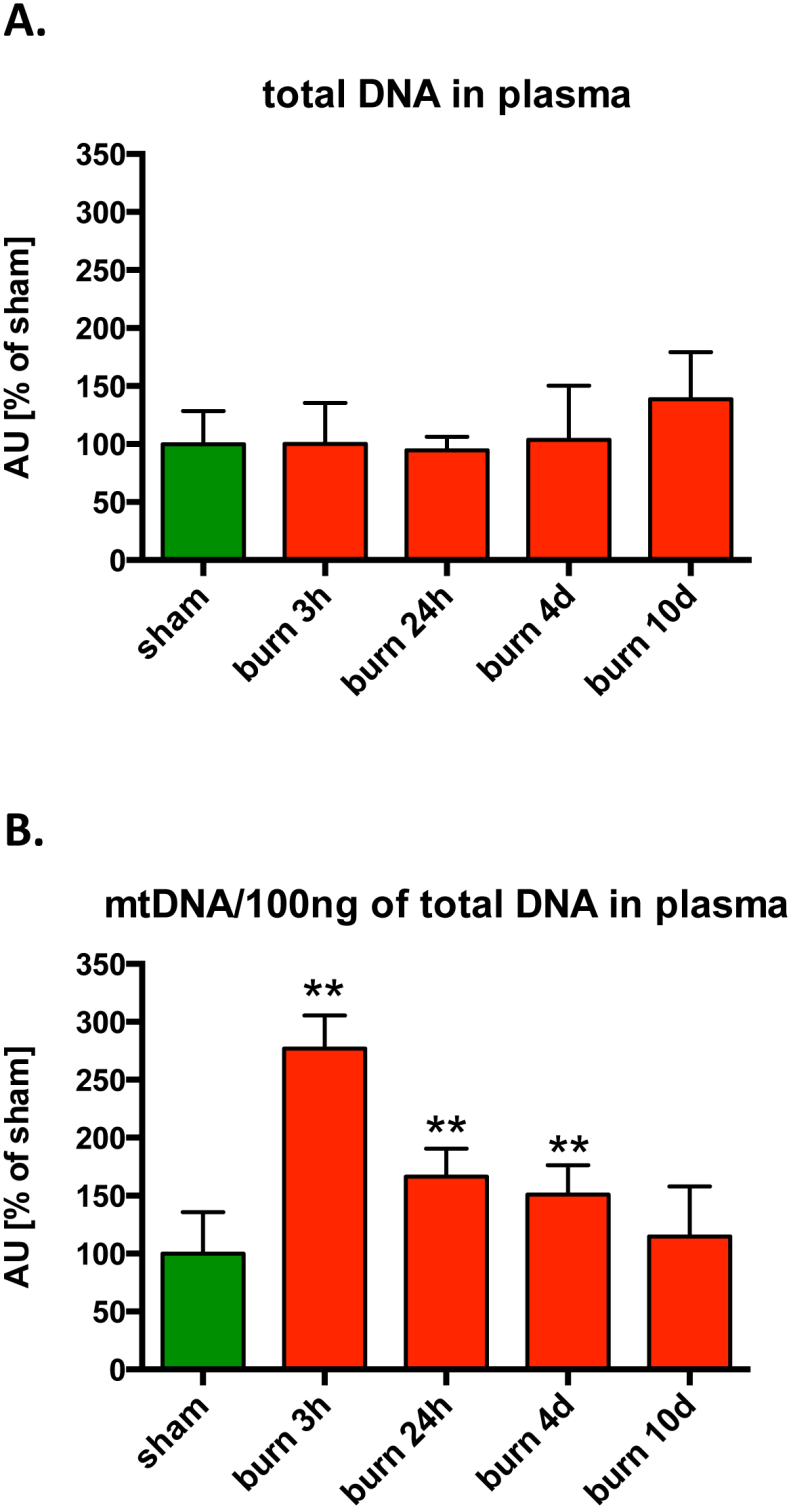
Changes in circulating total and mitochondrial DNA in response to burn injury. The amount of **(A)** total DNA and **(B)** mitochondrial DNA fragments in plasma of mice subjected to burn injury was analyzed using qPCR. The results are based on n = 6 per group and represent mean±SD. *, ** p<0.05 and p<0.01 respectively, relatively to sham treated animals.

Increased levels of cell-free nuclear DNA-derived fragments in the plasma of burn patients has recently been reported; this phenomenon was attributed to cell necrosis due to burn injury [13]. With the availability of real-time quantitative PCR technology, the presence of mitochondrial DNA in various human body fluids has also been associated with several pathologies including metabolic diseases, HIV and cancer [14–16]. Our analysis of the level of DNA damage in the various parenchymal tissues post-burn showed that mitochondrial DNA damage primarily occurs in the lung and heart tissue. We analyzed amounts of total and mitochondrial DNA in serum isolated from burn-injured animals at various time points. No significant differences were detected in the amounts of total DNA in plasma between sham and burn injured animals (Fig 8A). However, there was a 3-fold increase in mitochondrial DNA fragments in the plasma of animals subjected to burn as early as 3 hours post-burn, which persisted to at least 4 days post-burn and returned to normal (undetectable) levels by 10 days post-injury (Fig 8B).

**Fig 9:**
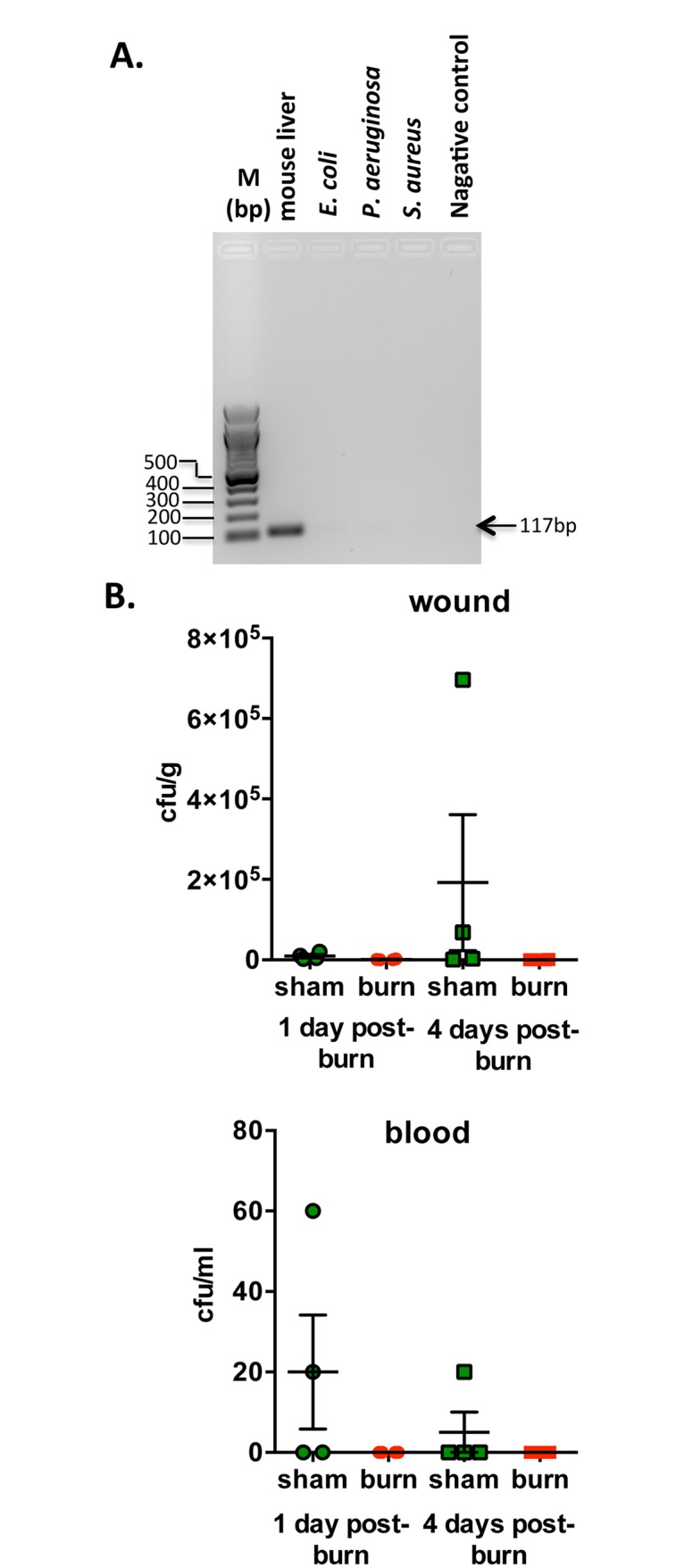
The current model of burn injury does not induce wound infections or bacteremia. (**A**) Specificity of mitochondrial DNA primers were tested using total DNA isolated from three common found in bacteremia/would infection strains of bacteria, *E*. *coli*, *P*. *aeruginosa*, and *S*. *aureus*. Lack of amplification of 117bp fragments indicates primers specificity toward mitochondrial DNA. (**B**) Bacterial cultures were performed using blood and burn wounds or sham-injured skin from mice at 1 and 4 days post-burn or sham injury. Graphs show individual cfu/g (wound/skin) or cfu/ml (blood) values, and means ± SD. N = 4 mice/group.

Since mitochondrial DNA resembles bacterial DNA, in order to assess the potential contribution of systemic bacterial infection to the pathogenesis of our mouse burn model, we tested mitochondrial DNA specific primers using DNA isolated from three strains of bacteria commonly found in infected wound, namely, *E*. *coli*, *P*. *aeruginosa*, and *S*. *aureus*. Amplification of 117bp DNA fragment was found only when total DNA of mouse liver was used as a template for PCR reaction (Fig 9A). To further test whether the results shown in the current study are due to burn injury per se, or, alternatively, are also related to complications induced by wound infection and bacteremia, wound and blood bacterial cultures were performed at 1 and 4 days post-burn or sham injuries (Fig 9B). Negligible amounts of bacteria were cultured from burn wounds at 1 and 4 days post-burn (1,100 and 1,600 cfu/g, respectively), and were well below those that are typically associated with wound infections (>10^5^ cfu/g). Bacterial counts in burn wounds were actually lower than in sham, non-injured skin, presumably due to destruction of normal skin flora during the scald burn. Additionally, there were no clinical signs of infection in burn-injured mice throughout the study, and blood cultures were also negative (Fig 9B).

### Burn Induces Infiltration of Macrophages and Neutrophils in Lungs and Heart

**Fig 10:**
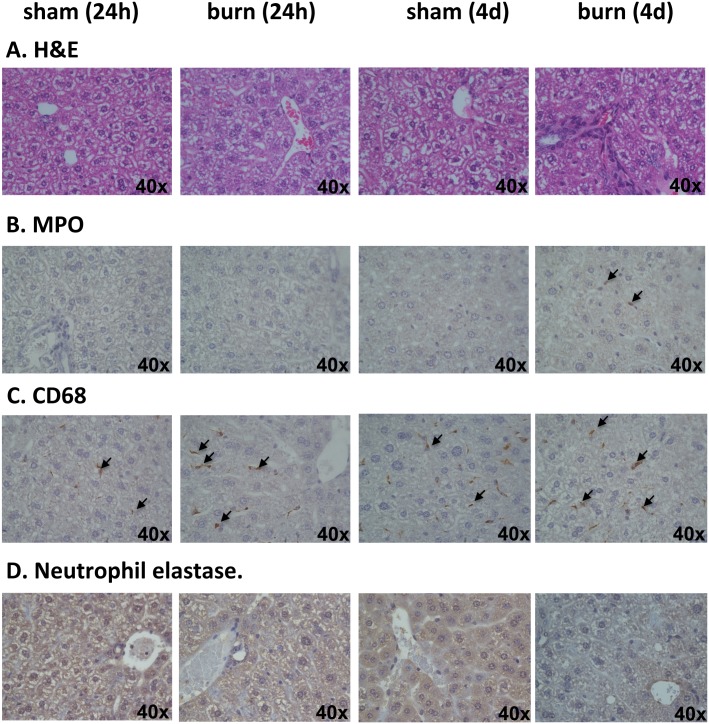
Changes in histopathology of liver induced by burn. Liver sections of animals at 1 and 4 days post burn injury with there control shams were stained with (**A**) H&E; (**B**) MPO; (**C**) CD68; (**D**) neutrophil elastase. Representative pictures of n = 4 for each animal group are shown.

**Fig 11:**
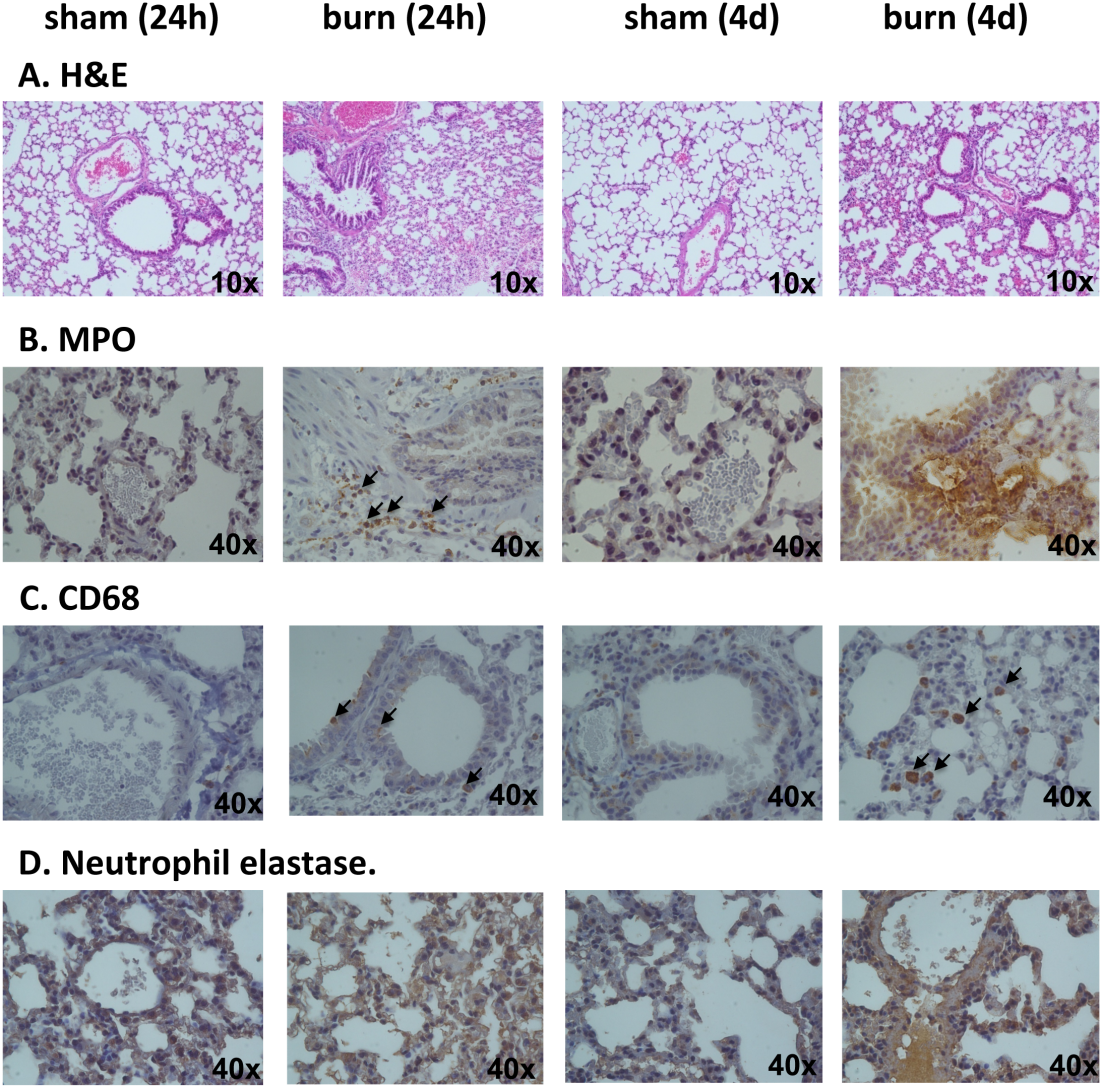
Changes in histopathology of lungs induced by burn. Lung sections of animals at 1 and 4 days post burn injury with there control shams were stained with (**A**) H&E; (**B**) MPO; (**C**) CD68; (**D**) neutrophil elastase. Representative pictures of n = 4 for each animal group are shown.

**Fig 12:**
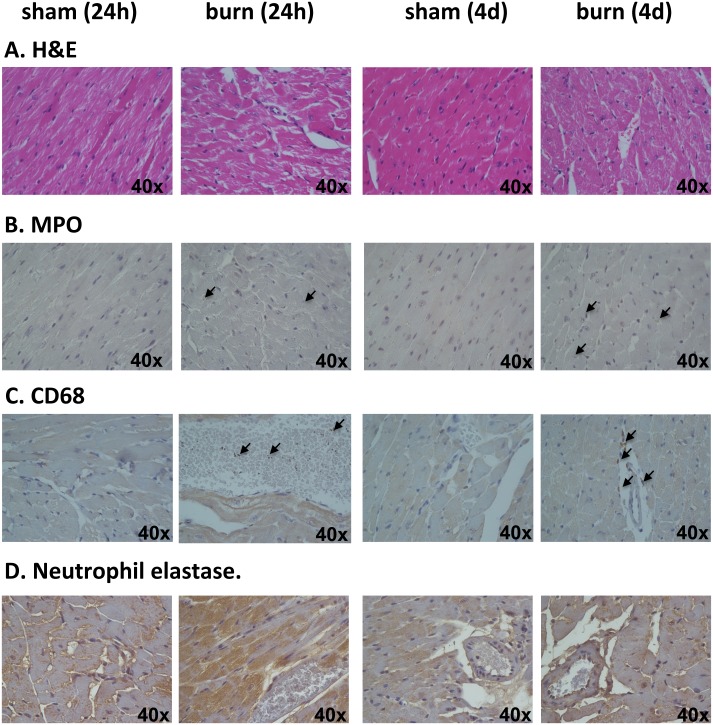
Changes in histopathology of heart induced by burn. Heart sections of animals at 1 and 4 days post burn injury with there control shams were stained with (**A**) H&E; (**B**) MPO; (**C**) CD68; (**D**) neutrophil elastase. Representative pictures of n = 4 for each animal group are shown.

Finally, we performed histopathology and immunohistochemistry analysis of liver (Fig 10), lung (Fig 11) and heart (Fig 12) tissue, using H&E staining and antibodies against myeloperoxidase (MPO), CD68 and neutrophil elastase. Burn-induced changes in tissue histopathology were observed in all tissues analyzed. Moreover, significant staining with MPO of the lung tissue (but not the liver and the heart) was noted at 4 days post burn (Fig 11B), supporting our biochemical analysis of MPO activity presented in Fig 7. In addition, we detected enhanced staining with CD68 in lungs (Fig 11C) and heart (Fig 12C), indicating that these tissues are affected not only by the infiltration of polymorphonuclear cells (neutrophils), but also by infiltration of cells from the monocytic/macrophage. In line with the MPO data, there was some basal staining for elastase in all tissues, which markedly increased after burn injury, with the most pronounced changes occurring in the lung (Figs 10D, 11D and 12D).

## Discussion

Our data indicate that burn injury in mice induces time-dependent and tissue-specific changes in mitochondrial bioenergetics, mitochondrial DNA damage and mitochondrial biogenesis that persist beyond the acute post-burn period (Table 1). Moreover, elevated levels of mitochondrial DNA fragments in the circulation suggest that mitochondria are not only targets but may also be effectors/transducers of the cellular alterations associated with burn injury. Moreover, our results suggest that the changes reported in the current study are related to burn injury per se (rather than a combination of burn and subsequent sepsis), since we found no evidence of infection, bacteremia, or circulating bacterial DNA.

**Table 1:** Changes in various parameters in various organs after burn injury. A single arrow shows a statistically significant change; double arrows show statistically significant changes that are more than 100% increases (i.e. doubling of the parameter) or a more than 50% decrease in the given parameter. Horizontal arrows indicate no significant change in the parameter.

		Basal respiration	Maximal respiratory capacity	Nuclear DNA integrity	Mitochondrial DNA integrity	Mitochondrial biogenesis	MDA	MPO	PARylation
**Liver**	**3 hours**	**→**	**↓**	**→**	**→**	**↓**	**↑**	**↓**	**→**
**Liver**	**4 days**	**↓**	**↑**	**→**	**→**	**→**	**↑**	**→**	**↓↓**
**Liver**	**10 days**	**↓**	**→**	**→**	**→**	**→**	**↑**	**→**	**↓↓**
**Lung**	**3 hours**	**↓**	**→**	**→**	**↓↓**	**↓**	**→**	**→**	**→**
**Lung**	**4 days**	not tested	not tested	**→**	**↓**	**↑**	**↓**	**↑**	**→**
**Lung**	**10 days**	↑	↑	**→**	**↓↓**	**↑**	**↑**	**↑↑**	**→**
**Heart**	**3 hours**	not tested	not tested	**→**	**→**	**→**	**↑↑**	**→**	**→**
**Heart**	**4 days**	not tested	not tested	**↓**	**↓↓**	**→**	**↑↑**	**→**	**→**
**Heart**	**10 days**	not tested	not tested	**↓**	**↓**	**→**	**↑↑**	**→**	**↑↑**

One of the primary aims of the current study was to examine the effect of burn injury on several mitochondrial functions in two parenchymal organs, the liver and the lung. We have studied the liver in more detail in terms of temporal resolution (3 and 24 days after burn injury, as well as 4, 10, 20 and 40 days). At the earliest time point (3 hours post-burn), most mitochondrial parameters remained unchanged, but 24 hours after burns a significant suppression of most mitochondrial parameters (including basal respiration and maximal respiratory capacity) occurred. Surprisingly, these alterations were maintained for 10 days post-burn. Some of the parameters normalized by 20 and 40 days, but even at the latest time point studied (40 days) basal respiration continued to show a significant elevation compared to baseline (Fig 1). Mitochondria isolated from the lung tissue presented with a similar pattern; an early suppression (detected at 3 days), followed by a later increase (detected at 10 days). However, in the lung tissue, at 10 days, the mitochondrial function was not only normalized, but markedly elevated compared to the initial control values (Fig 2). The delayed increase in lung mitochondrial function (but less increase in liver mitochondrial function) however, is not explained by our findings of mitochondrial biogenesis (Fig 5), since in the Seahorse experiments we plate a standard amount of mitochondria, and therefore the Seahorse method specifically measures the intrinsic changes in the function of the mitochondria, but it is not affected overall amount of mitochondria present in the tissue.

The conclusion that changes in the mitochondrial function following burn show a time-dependent and tissue-dependent pattern is further supported by a recent study investigating changes in the metabolic function of white adipose tissue. In this tissue, the early time points post burn injury were not associated with a significant suppression of mitochondrial function, while later time points (10 days to 40 days) were associated with an increase in mitochondrial respiration, as well as an increase in mitochondrial biogenesis (assessed by the measurement of citrate synthase activity) [17]. It is also important to mention that in the white adipose tissue, the later time points were also associated with an upregulation of UCP1, a hallmark of 'browning' (conversion of white adipose tissue to metabolically more active brown adipose tissue) [17].

Mitochondrial DNA damage has been implicated in a wide variety of pathophysiological conditions, including various forms of critical illness [18–20]. The current report indicates that the degree of mitochondrial (and, to some extent, nuclear) DNA damage is highly tissue dependent. For instance, in the liver, mitochondrial (or nuclear) DNA integrity was well maintained after burn injury (even though the function of the mitochondria was significantly affected). In contrast, in lung tissue mitochondrial DNA was already damaged by 3 hours, and the damage was sustained even at 10 days after burn. The heart tissue responded similarly to the lung tissue with respect to mitochondrial DNA damage; however, in the heart tissue there was also observed significant damage to the nuclear DNA (a change not observed in the other two organs analyzed). DNA damage can be viewed as a deleterious consequence of burn injury, even though, according to the current study, it does not closely correlate with the changes in mitochondrial bioenergetic function. For example, in the liver tissue, there was a marked suppression of mitochondrial function at 1–3 days post-burn, even though we could not detect any changes in mitochondrial DNA integrity in the same tissue. Although it is an attractive hypothesis that mitochondrial DNA damage during burn injury suppresses the transcription of key mitochondrial electron transport chain proteins, which, in turn, impairs mitochondrial function, we must conclude that changes in mitochondrial DNA integrity are probably not the primary drivers of mitochondrial dysfunction in the current experimental model.

Oxidative/nitrative stress has been implicated in the pathogenesis of burn injury by multiple studies [5,21–29]. Since oxidative/nitrative stress is also known to induce DNA damage and mitochondrial dysfunction, it is a reasonable hypothesis that the production of reactive oxidants and free radicals is a significant causative factor in the changes in mitochondrial function and mitochondrial DNA integrity observed in the current study. The measurement of tissue malondialdehyde levels (which results from lipid peroxidation of polyunsaturated fatty acids) is only one of many measures of oxidative stress, and, therefore, it must be interpreted with caution. Nevertheless, MDA analysis has been widely used in studies focusing on tissue damage during critical illness, and it tends to correlate with oxidative stress 'load' in various organs. The results of the MDA analysis in the current study only show a partial correlation with the mitochondrial bioenergetic alterations and the degree of DNA damage in the various tissues. For example, in the liver tissue, MDA was elevated both at the early post-burn time points (where mitochondrial function was found to be suppressed) and at the later time points (where mitochondrial function returned to normal even in the absence of increased mitochondrial biogenesis). Furthermore, this increased oxidative stress 'load' in the liver was not associated with detectable mitochondrial or nuclear DNA damage. On the other hand, in the lung tissue, for most of the time points analyzed, the marked alterations in bioenergetics and the significant damage to the mitochondrial DNA occurred in the absence of detectable increases in MDA levels. Notably, we have observed the most pronounced increases in MDA elevation in the heart tissue, and this was the organ with indiscriminate DNA damage (i.e. damage to both nuclear and mitochondrial DNA). This correlation may suggest a causative relationship, although this issue remains to be directly evaluated (e.g. by testing the effect of antioxidants and free radical scavengers on mitochondrial and nuclear DNA integrity) in the current model.

Previous preclinical and clinical studies have demonstrated increases in various parenchymal tissues as well as in circulating leukocytes after burn injury [5,30–33]. Importantly, changes in tissue PARP activity post-burn (as assessed by the detection of tissue levels of PAR polymers) mirrored the changes in nuclear DNA damage: PARP activation was observed in the tissue where nuclear DNA damage was observed, while in the two other tissues, where nuclear DNA damage was not noted, increases in tissue PARylation were not noted, either. Although PARP1 has both nuclear and mitochondrial localization in many cell types [34–37], the majority of cellular PARylation is of nuclear origin, and the current findings are also consistent with this notion. Interestingly, in the liver, PARylation *decreased* at later time points post-burn. PARP1 can be degraded by caspases and other intracellular proteases (including metalloproteinases) [34,35,38] and caspase activation has previously been reported in the liver post-burn [39,40]. We hypothesize that PARP1 degradation may be responsible for the observed decrease in PAR polymers in the liver after burn injury; further studies detecting caspase activity, full-length PARP1 as well as cleaved PARP fragments are necessary to further explore this hypothesis.

In many models of critical illness, elevations in tissue levels of MDA (a marker of oxidative stress) and MPO (myeloperoxidase, a marker of inflammatory cell infiltration) or macrophage/neutrophil infiltration occur hand-in-hand [25–28]. It is generally assumed that these two phenomena are part of interrelated pro-oxidant / pro-inflammatory processes, whereby increased oxidative stress activates pro-inflammatory signaling pathways, which, in turn, recruit mononuclear cells into the oxidatively damaged tissues, which, in turn, create further oxidative/nitrative damage, tissue injury and further pro-inflammatory signaling. However, in the current experimental model, changes in MDA and MPO showed different temporal and tissue-specific patterns, indicative of a more complex relationship between these two parameters. For example, the increased MDA levels in the liver were not associated with increased MPO levels at any of the time points studied. In contrast, in the lung, there was a time-dependent increase in MPO (which was the most pronounced at the latest time point studied), even though in this tissue we were unable to detect any increase in MDA at any of the time points investigated. In the heart, MDA levels showed a marked initial increase, followed by a gradual decrease, while MPO levels showed the opposing trend (no change in the early time points, followed by an increase in the latest time points studied). Overall, the changes in MDA and MPO did not show a good correlation with either the changes in mitochondrial bioenergetic parameters, nor with the degree of mitochondrial or nuclear DNA integrity in any of the organs studied.

One additional parameter that we have studied in the current set of experiments was the presence of circulating DNA (total DNA as well as mitochondrial DNA). While the amount of total DNA was unaffected by burns, the relative proportion of mitochondrial DNA was increased at the earliest time point (3 hours), followed by a rapid decline, reaching baseline control levels by 10 days post-burn. While this pattern of circulating mitochondrial DNA levels did not closely correlate with any of the parameters studied in the present report (bioenergetic parameters, DNA damage, tissue MDA or MPO), the pattern is similar to that of many cytokines and other pro-inflammatory mediators that show an initial increase in various forms of critical illness, followed by a time-dependent decline. Another potential explanation may be that due to presence of DNases in blood, the mitochondrial DNA released at the earlier time point post burn injury is subject to a subsequent degradation process. Nevertheless, circulating mitochondrial DNA has been identified as one of the mediators that may contribute to remote organ injury in various forms of critical illness [18–20] and the current findings are also in line with such a potential role in the current model.

Taken together, the current data are consistent with the hypothesis that burn induces a systemic response, which includes time-dependent and organ-specific changes in mitochondrial function and mitochondrial DNA integrity. These changes do not show a close correlation, either with tissue levels of oxidative stress or inflammatory cell infiltration, nor with the amount of mitochondrial DNA in the circulation, suggesting the presence of multiple causative factors and complex interrelationships between the various parameters studied here. The current series of experiments, although by design descriptive and not mechanistic, further support prior data showing that burn induces a multi-organ dysfunction [1–4], that burn induces a multi-organ dysfunction [1–4], that burn induces oxidative/nitrative stress [5–7,21,23,24], that burn induces mononuclear and polymorphonuclear cell infiltration into various tissues [5,41–47], that burn can induce PARP activation [5,30–33] and that burn can impair mitochondrial function [8,48–53]. Nevertheless, to our knowledge, the current study represents the most comprehensive analysis to date of multiple mitochondrial parameters in a murine model of burn injury. We are well aware of an on-going, intensive discussion on the potential utility (or lack thereof) of murine models to study human critical illness; with the current status of the discussion being that—while much caution and many caveats remain to be considered—murine models remain useful for basic and applied research into the pathogenesis of human disease [54–57]. Taken together, we hope that the current findings will serve as a good starting point for future, mechanistic studies to elucidate the role of various (local and circulating) factors and mechanisms in the pathogenesis of the profound alterations in mitochondrial function induced by burns.
